# Hypertonia of the Big Toe Revealing Parkinson’s Disease: A Case Report

**DOI:** 10.7759/cureus.58203

**Published:** 2024-04-13

**Authors:** Houssam Mahla, Abdelilah Rhoul, Mohammed Gartit, Souhail Yachaoui, Ahmed Amine EL Oumri

**Affiliations:** 1 Physical Medicine and Rehabilitation, Mohammed VI University Hospital, Oujda, MAR; 2 Medicine and Pharmacy, Mohamed I University, Oujda, MAR; 3 Physical Medicine, Mohammed VI University Hospital, Oujda, MAR; 4 Medicine, Mohamed I University, Oujda, MAR

**Keywords:** parkinson’s disease, focal dystonia, hypertonia of the hallux, striatal foot, striatal deformities

## Abstract

Despite being less commonly discussed than other motor symptoms such as tremors and bradykinesia, hypertonia of the hallux holds diagnostic and prognostic significance in Parkinson’s disease (PD). This motor anomaly is dissected within the context of the broader clinical spectrum of PD symptoms, emphasizing its importance alongside its cardinal symptoms. This case report underscores the importance of accurate clinical assessment especially thorough neurological evaluation in discerning hallux hypertonia, potentially enabling early disease recognition and intervention. By synthesizing these clinical insights, we trust that this case report contributes to an enhanced understanding of hypertonia of the hallux as a distinctive clinical presentation in PD fostering improved diagnostic precision.

## Introduction

Hypertonia is defined as abnormally increased resistance to externally imposed movement about a joint [1]. This can be caused by various conditions such as spasticity, dystonia, or rigidity, resulting in unusual postures, including foot deformities like striatal foot (SF), which is frequently observed in individuals with advanced Parkinson's disease (PD) [2]. These deformities could be developed not only in the later stages of PD but also in the initial phases of the disease and other Parkinsonian disorders [3]. The likelihood of misdiagnosing SF deformities is notable, especially when they appear early in the absence of the cardinal signs of PD such as tremors [3]. They could also be mistaken as primary dystonia, whether sporadic or hereditary, despite the fact that this condition rarely affects the feet [4]. This case report highlights the importance of a complete clinical assessment and the consideration of PD in front of isolated toe dystonic cases.

## Case presentation

A 75-year-old woman presented to our Physical Medicine and Rehabilitation Department. Her chief complaints were walking difficulties that had started insidiously one year ago with a shuffling gait pattern and frequent trips (Video 1). She reported a personal history of hypertension, with no personal or familial history of neurologic disorders.

**Video 1:** PD symptoms in our patient Video showcasing PD symptoms in our patient: short-stepped gait, loss of arm swing, forward-trunk flexion, and limb rigidity PD, Parkinson's disease

**Figure 1:**
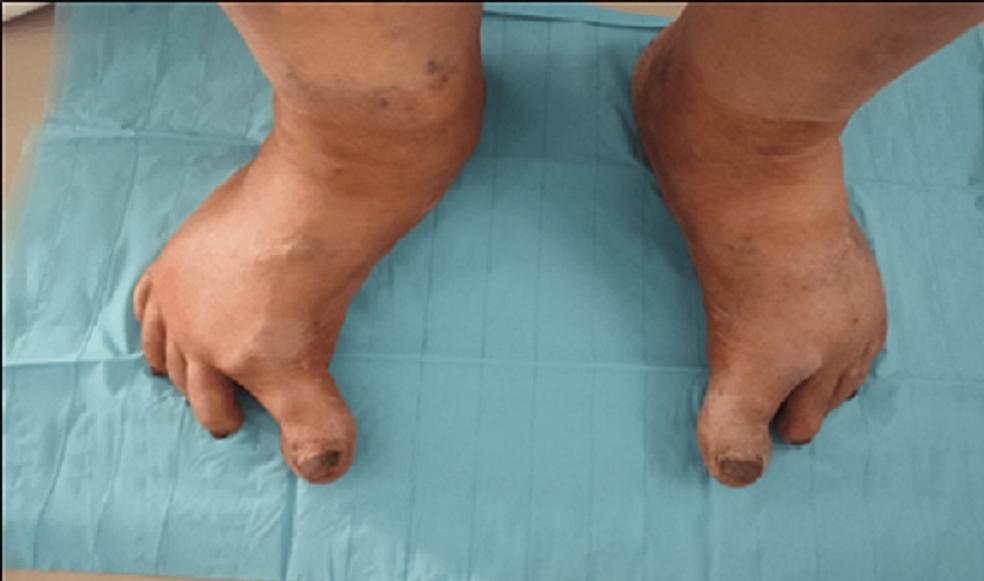
Bilateral hallux hypertonia more pronounced on the right foot

An initial clinical examination revealed a hypertonia of her big toes that was more significant on the right foot (Figure 1). This observation prompted us to conduct a more thorough neurological examination proving an akinetic-rigid syndrome with gait disturbance and axial rigidity. Upon evaluation, the UPDRS Part 3 score was determined to be 49, reflecting moderate to severe motor symptomatology. This assessment was indicative of substantial disease progression, as the Hoen and Yahr stage reached three, highlighting moderate to severe functional impairment. Additionally, the PDQ-39 score of 25.7 revealed a moderate decline in PD-related quality of life.

**Figure 2:**
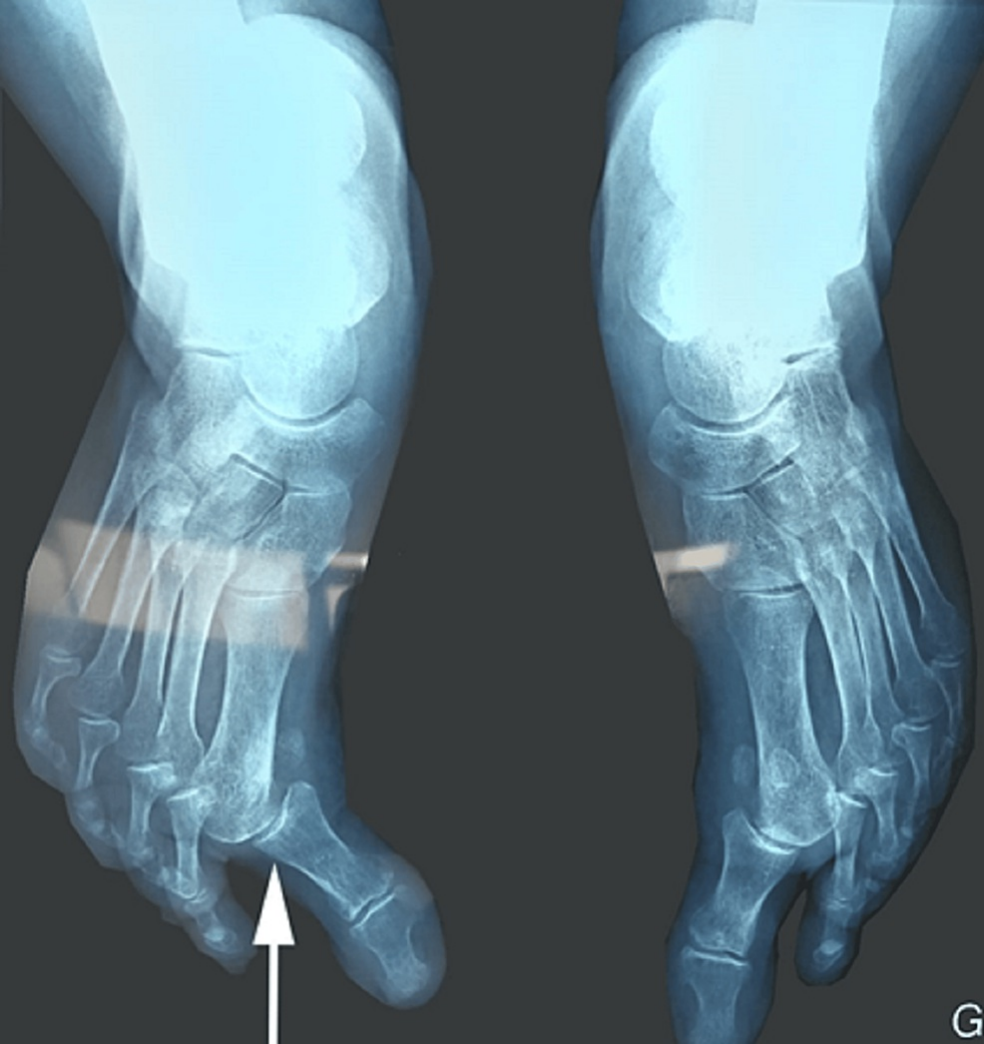
An anteroposterior standard foot radiograph showing bilateral dislocation of the first MTP joint more significant on the right (white arrow) MTP, metatarsophalangeal

Furthermore, standard X-ray imaging showed a bilateral dislocation of the first metatarsophalangeal (MTP) joint more significant on the right (Figure 2). Additionally, a cerebral CT scan was performed and showed no abnormalities. Biological tests for rheumatic conditions were conducted, encompassing tests for rheumatoid factor and anti-cyclic citrullinated peptides antibodies (anti-CCP), both yielding negative results.

Confronted with this clinical presentation and the absence of any anomalies detected in the cerebral CT scan, we concluded the diagnosis of PD associated with focal dystonia. We recommended physical therapy sessions based on gait and balance training. The possibility of toxin injection for her big toes’ dystonia will be evaluated after the completion of the rehabilitation sessions. The patient was then referred to the neurology department for complementary management. 

## Discussion

Several dystonic manifestations were reported in PD affecting different body parts, including focal and axial forms. SF is a focal dystonia form with a typical feature described as having a big toe extension, flexion of the other toes, equinovarus foot, and pain. This dystonic phenomenon could also be limited to the big toe only, realizing the striatal toe deformity which is the SF deformity without the equinovarus foot posture [5]. These striatal deformities were reported in both advanced stages of PD and initial symptoms of PD [3]. In our case, the patient presented an equinovarus foot with big toe adduction without claw feet deformities.

The prevalence of SF has not been systematically studied, with reports of 20% to 40% of foot dystonia development in PD patients receiving levodopa treatment [3]. Nevertheless, approximately 2.4% of individuals within the PD population experience dystonia prior to treatment initiation [6]. Furthermore, several studies reported different types of dystonic features including SF in untreated PD patients and even before the onset of parkinsonism [7-9]. In addition, dystonia is more likely related to the young onset of PD rather than late-onset PD [10].

SF is not a specific symptom of PD; other causes could also lead to this deformity notably rheumatologic conditions such as rheumatologic arthritis (RA). In RA conditions, the great toe typically develops hallux valgus deformation characterized by subluxation of the phalanx at the MTP joint of the other toes that predominantly occurs dorsally [11]. The context and clinical examination, in addition to paraclinical tests, generally help to guide the diagnosis. Furthermore, striatal toe deformity could also be seen in post-stroke spastic patients when the extensor hallucis longus muscle is affected [12]. Other disorders with similar foot deformities including progressive supra-nuclear palsy, peripheral vascular disease, and lumbar canal stenosis were also reported [3].

Different management strategies for PD-related dystonia including pharmacological and procedural modalities were reported. Anti-parkinsonism drugs have different degrees of response with positive results being reported; nevertheless, the response seems to be less predictable [13,14]. Positive results with the use of anti-cholinergic and baclofen were also published [15]. Botulinum toxin (BT) is another therapy to treat focal dystonia [16]. Giladi reported positive results after injecting BT in the hallucis muscle of three patients [17]. Several other studies supported the efficacity of the BT in the treatment of focal dystonia in PD especially in cervical and foot dystonia [18,19]. An ongoing double-blinded randomized clinical trial (RCT) aimed to assess the safety and efficacity of the injection of BT in PD patients with foot dystonia (NCT04277247). The results from this RCT will highlight the role of this therapy in the management of SF.

Other therapies were also proposed, particularly deep brain stimulation which was used in segmental and generalized dystonia. However, high-evidence studies are still lacking with this therapeutic option [20].

## Conclusions

In conclusion, while our case highlights the importance of considering PD in patients with atypical motor symptoms like hypertonia of the big toe, the findings are limited by the nature of our study. Future studies involving larger case series studies are needed to confirm the prevalence and clinical significance of hallux hypertonia as an early or atypical manifestation of PD.
